# Extended Reality-Based Head-Mounted Displays for Surgical Education: A Ten-Year Systematic Review

**DOI:** 10.3390/bioengineering11080741

**Published:** 2024-07-23

**Authors:** Ziyu Qi, Felix Corr, Dustin Grimm, Christopher Nimsky, Miriam H. A. Bopp

**Affiliations:** 1 Department of Neurosurgery, University of Marburg, Baldingerstrasse, 35043 Marburg, Germany; felix.corr@uk-gm.de (F.C.); dustin.grimm@uk-gm.de (D.G.); nimsky@med.uni-marburg.de (C.N.); 2 Department of Neurosurgery, First Medical Center of Chinese PLA General Hospital, Beijing 100853, China; 3 Center for Mind, Brain and Behavior (CMBB), 35043 Marburg, Germany

**Keywords:** extended reality, education, surgery, augmented reality, mixed reality, virtual reality, knowledge, skill, simulation, residents, head-mounted display

## Abstract

Surgical education demands extensive knowledge and skill acquisition within limited time frames, often limited by reduced training opportunities and high-pressure environments. This review evaluates the effectiveness of extended reality-based head-mounted display (ExR-HMD) technology in surgical education, examining its impact on educational outcomes and exploring its strengths and limitations. Data from PubMed, Cochrane Library, Web of Science, ScienceDirect, Scopus, ACM Digital Library, IEEE Xplore, WorldCat, and Google Scholar (Year: 2014–2024) were synthesized. After screening, 32 studies comparing ExR-HMD and traditional surgical training methods for medical students or residents were identified. Quality and bias were assessed using the Medical Education Research Study Quality Instrument, Newcastle–Ottawa Scale-Education, and Cochrane Risk of Bias Tools. Results indicate that ExR-HMD offers benefits such as increased immersion, spatial awareness, and interaction and supports motor skill acquisition theory and constructivist educational theories. However, challenges such as system fidelity, operational inconvenience, and physical discomfort were noted. Nearly half the studies reported outcomes comparable or superior to traditional methods, emphasizing the importance of social interaction. Limitations include study heterogeneity and English-only publications. ExR-HMD shows promise but needs educational theory integration and social interaction. Future research should address technical and economic barriers to global accessibility.

## 1. Introduction

Surgical education is characterized by the need for residents and medical students to acquire a broad range of knowledge and skills within a limited time frame [1]. This training emphasizes hands-on, interactive learning, requiring high levels of engagement and focus. However, high workload constraints have significantly reduced opportunities for practical training, and ethical concerns arise from the potential discomfort and risk to patients during training procedures [2,3,4]. These challenges necessitate the exploration of innovative educational technologies to enhance training outcomes [5,6,7,8,9,10].

Extended reality (ExR) technologies encompass virtual reality (VR), augmented reality (AR), and mixed reality (MR) and have emerged as promising tools for surgical education [11]. These technologies create immersive, interactive simulations tailored to specific educational needs, offering a risk-free environment for trainees to practice procedures and hone their skills [6,7,8,9,10]. Implemented mainly through head-mounted displays (HMDs), ExR technologies provide several technical advantages, including lower cost, easier accessibility, higher levels of immersion, improved spatial awareness, and more intuitive interaction capabilities [12,13,14,15,16,17,18]. These advantages have sparked research interest in the potential benefits of HMDs in surgical education, reflected in the steady increase in studies on this topic [19].

ExR provides immersive, engaging learning experiences that can improve knowledge retention and skill acquisition [20]. Based on Fitts and Posner’s motor skill acquisition theory [21], ExR supports all phases of learning psychomotor surgical skills by allowing repetitive practice and immediate feedback, facilitating the transition from conscious effort to automatic execution [22]. Furthermore, ExR can potentially improve health systems in non-high-income countries by making advanced training tools more accessible [23,24,25].

Despite the growing body of research, the effectiveness of ExR in surgical education and its theoretical underpinnings remain insufficiently understood [26,27,28,29]. Most existing reviews on ExR-HMDs focus on technological trends rather than educational outcomes or theories, illustrated in Table 1. This review aims to evaluate the effectiveness of ExR-HMD technology in surgical education by examining its impact on educational outcomes, identifying its strengths and limitations from a pedagogical perspective, and exploring how educational theories can inform the design and implementation of surgical training programs using ExR-HMDs.

To achieve this, the research focuses on three analytical questions (AQs) under three dimensions based on the Technological Pedagogical Content Knowledge (TPACK) model [30]:

- **AQ 1. Content:** What types of surgical education content and activities are involved?
- **AQ 2. Pedagogy:** What are the observed learning outcomes and effectiveness, particularly regarding knowledge acquisition, skill development, and attitudinal changes, and what pedagogical theories are employed in surgical training?
- **AQ 3. Technology:** What types of ExR-HMDs are utilized, and what are their advantages and disadvantages in surgical education?

## 2. Methods

This systematic review analyzes and synthesizes the selected studies following the Cochrane Handbook for Systematic Reviews of Interventions and reports the findings adhering to the Preferred Reporting Items for Systematic reviews and Meta-Analyses (PRISMA) guidelines (see Appendix A) [31,32]. The study was registered in PROSPERO with registration number CRD42024533114.

Section 2.1 outlines the eligibility criteria and search strategy. Section 2.2 details the search and screening process. Section 2.3 describes the evidence extraction process. Section 2.4 explains the tools used to assess the included studies’ quality and bias. Finally, Section 2.5 describes the synthesis of the data.

### 2.1. Eligibility Criteria and Search Strategy

#### 2.1.1. Eligibility Criteria

The literature published between 2014 and 2024 presenting findings on using ExR-HMDs in surgical education was considered. The criteria employed for the comprehensive review of these articles are detailed in Table 2, following the Population, Intervention, Comparison, Outcomes, and Study (PICOS) framework [33].

#### 2.1.2. Data Source

Seven electronic databases encompassing medicine and computer science, i.e., PubMed (https://pubmed.ncbi.nlm.nih.gov/, accessed on 30 March 2024), Cochrane Library (https://www.cochranelibrary.com/, accessed on 30 March 2024), Web of Science (https://www.webofscience.com/, accessed on 30 March 2024), ScienceDirect (https://www.sciencedirect.com/, accessed on 30 March 2024), Scopus (www.scopus.com, accessed on 30 March 2024), ACM Digital Library (https://dl.acm.org/, accessed on 30 March 2024), and IEEE Xplore (https://ieeexplore.ieee.org/, accessed on 30 March 2024), were accessed via the instituional portal to retrieve articles or materials in English. Additionally, WorldCat (https://search.worldcat.org/, accessed on 30 March 2024) and Google Scholar (https://search.worldcat.org/, accessed on 30 March 2024) as databases for grey literature were queried to complement the search and minimize omissions, adopting a strategy similar to that of Haddaway et al. [34] and Barteit et al. [26], capturing the first 100 or 200 most relevant results, respectively.

#### 2.1.3. Search Terms and Syntax

Three conceptual groups were initially established to ensure coverage of the literature potentially related to the topic: ExR (including VR, AR, and MR), surgery, and education. Keywords and synonyms were then defined based on several preliminary informal searches, as illustrated in Table A1.

Boolean operators, such as AND and OR, were used to link search terms. To balance comprehensiveness and precision, both controlled vocabulary (subject headings) and free-text terms were searched, with wildcards (*) employed to cover as many grammatical variants as possible. Where supported by the database, searches were preferentially confined to titles, abstracts, and keywords. The defined generic search syntax was as follows:

- (“virtual reality” OR “augmented reality” OR “mixed reality” OR “extended reality”) AND ((surg* OR operat* OR procedur*) AND (educat* OR teach* OR train* OR learn*))

Finally, the search string was adjusted to conform to the syntax specific to each data source, as shown in Table A2.

### 2.2. Search and Screening Execution

#### 2.2.1. Search Execution

The first author conducted the formal query on 30 March 2024. The databases yielded extensive results, as detailed in Table A3, due to the inclusion of all types of ExR, VR, AR, and MR technologies, not just limited to HMDs. The records were downloaded and imported into Rayyan’s open-source reference management platform (https://www.rayyan.ai/, accessed on 30 March 2024) for further processing. Rayyan’s parser automatically detected duplicates, which were then manually removed.

#### 2.2.2. Title and Abstract-Based Screening

The first author performed an “over-inclusive” title and abstract-based screening to ensure a broader selection at this preliminary stage. Publications with obviously irrelevant topics and materials, such as reviews, letters, anecdotes, and non-English publications, were excluded. Given the large volume of records, Rayyan’s built-in artificial intelligence rating system was utilized to expedite the screening process; however, it is important to note that all screening underwent manual review. The full texts were subsequently attempted to be accessed through the institutional portal.

#### 2.2.3. Full-Text Screening

Three reviewers independently applied the eligibility criteria to select studies for inclusion, ensuring a thorough and unbiased selection process. The reviewers independently screened the records for inclusion. Each reviewer’s decisions were initially made independently to maintain objectivity. Any conflicts between reviewers’ decisions were resolved through discussion and consensus, involving a fourth reviewer if needed. The decisions and the selection process were documented using the Rayyan platform.

#### 2.2.4. Extended Retrieval

To minimize the risk of omission, a snowball search (forward and backward) was conducted as per the guidelines of Wohlin et al. [35]. Additionally, references from related systematic reviews on similar topics were scanned.

### 2.3. Data Extraction

The extraction included data on bibliographic information, study design and methodology, participant demographics, and measures of effect as applicable. All three reviewers were involved in the data extraction process to ensure accuracy and reliability, with two extracting the data and one verifying it. Disagreements in data extraction were resolved through discussion between the three reviewers, involving a fourth reviewer if needed. Attempts were made to contact study investigators for missing or unclear data, helping to obtain a complete dataset for the review. Extracted data were systematically recorded in a structured format using SRDR+ (https://srdrplus.ahrq.gov/, accessed on 30 March 2024, version: 2019) and Microsoft Excel (Microsoft 365 MSO, Version 2406 Build 16.0.17726.20078, 64 Bit).

### 2.4. Quality and Bias Assessment

In assessing the risk of bias and quality of studies within this systematic review, particularly for the use of ExR-HMDs in surgical education, the focus was on key characteristics, including randomization methods, group allocation, and blinding procedures. The assessment was conducted at methodological levels to comprehensively understand potential biases.

Formal tools used for this risk of bias assessment included the Medical Education Research Study Quality Instrument (MERSQI) for evaluating the overall quality of medical education studies [36], the Newcastle–Ottawa Scale-Education (NOS-E) for non-randomized studies [37], and the Cochrane risk of bias tool for randomized trials (RoB 2) [38]. Additionally, the Risk Of Bias In Non-randomized Studies-of Interventions (ROBINS-I) tool was used to evaluate the internal validity of included non-randomized studies [39].

All three reviewers independently evaluated each study to manage this assessment. Any disagreements between them were resolved through discussion and consensus. If necessary, a fourth reviewer was consulted to mediate and help make a final decision, ensuring a rigorous and unbiased assessment process.

### 2.5. Data Synthesis

For this systematic review, the data synthesis adhered to the Synthesis Without Meta-analysis (SWiM) guidelines [40], tailored by the research question to categorize findings based on the impact of ExR-HMDs on acquiring knowledge and skills. The data organization was refined to reflect the distinct emphases on knowledge and skills within various medical specialties without employing a uniform metric for standardization. The studies meticulously extracted information pertinent to knowledge, skill enhancement, and specifics about the ExR-HMDs.

An evaluation of bias risk in non-randomized studies was conducted to ensure no significant concerns, leading to an unbiased synthesis of all eligible studies, each accorded equal importance. The study designs were inclusive and diverse, precluding a meta-analysis due to the expected variability in the data regarding knowledge and skill improvements. Consequently, effectiveness was collated from the individual reports of the studies.

## 3. Results

This section includes a summary of the included studies’ characteristics, quality assessments, and findings. Section 3.1 details the literature search and screening process, including reasons for exclusion, and reports the basic characteristics of the studies. Section 3.2 presents the quality assessment results of the studies and analyzes potential biases. Subsequently, Section 3.3, Section 3.4 and Section 3.5 address the research questions posed in the Section Introduction from three dimensions: content, pedagogy, and technology. In terms of content, Section 3.3 synthesizes the study results across six aspects: educational topics, target audience, grouping, traditional teaching methods, ExR-assisted teaching methods, and educational assessments. In terms of pedagogy, Section 3.4 summarizes the knowledge, skills, and attitude outcomes reported in the included studies and the educational theories cited. In terms of technology, Section 3.5 summarizes the characteristics of the ExR-HMDs used and outlines the benefits and drawbacks of ExR-HMDs from the perspectives of both trainees and educators.

### 3.1. Study Characteristics

**Figure 1:**
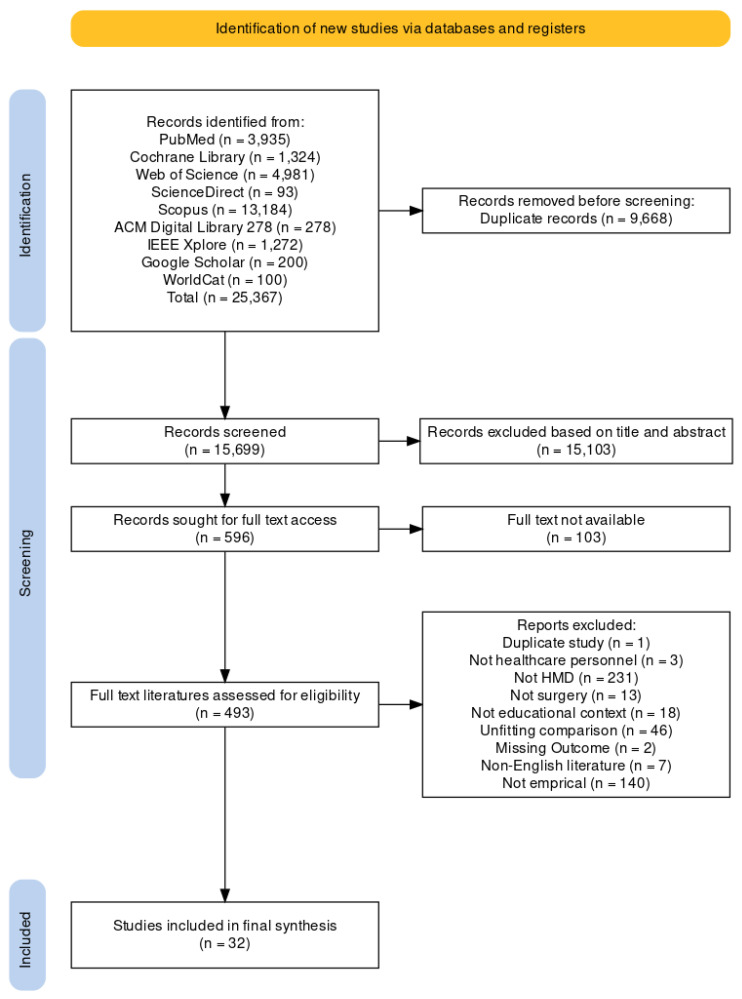
Flow diagram of PRISMA (The Preferred Reporting Items for Systematic reviews and Meta-Analyses) via online tools developed by Haddaway et al. [73].

Following the search strategy, 25,367 records were identified initially. After removing duplicates (n = 9668), 15,699 articles remained for title and abstract screening (see Table A3). This screening process yielded 596 potentially relevant articles, of which 493 met the criteria for full-text review. Using the PICOS criteria, an independent blinded evaluation initially selected 36 articles. No additional relevant papers meeting the criteria were identified during extended retrieval. During the data extraction phase, four articles that appeared to meet the inclusion criteria were excluded, resulting in a final inclusion of 32 studies. Figure 1 illustrates the screening and review process.

According to the PRISMA recommendations, the reasons for excluding four publications in the final stage are reported as follows: Ropelato et al. [41], where both ExR and traditional groups used the same AR simulator, differing only in task sequence; Silvero et al. [42], which reported only user experience and satisfaction without any learning outcomes; Wise et al. [43], a conference abstract that duplicated a study already included as a journal article from the same research group; and Barré et al. [24], which focused on cognitive load and psychological demands in training for VR and traditional groups without reporting any learning outcomes.

The included studies were published between 2014 and 2024. Most studies were conducted in the United States (n = 9) [44,45,46,47,48,49,50,51], followed by China (n = 6) [52,53,54,55,56,57], the United Kingdom (n = 5) [58,59,60,61,62], Canada (n = 3) [63,64,65], Germany (n = 2) [46,66], France (n = 1) [67], Denmark (n = 1) [68], the Netherlands (n = 1) [69], Greece (n = 1) [70], India (n = 1) [71], Pakistan (n = 1) [72], and Portugal (n = 1) [72]. According to the World Bank Group’s country classifications by income level, 24 studies (75.0%) were published in high-income countries [44,45,46,47,48,49,50,51,58,59,60,61,62,63,64,65,66,67,68,69,70,70], six studies (18.8%) in upper-middle-income countries [52,53,54,55,56,57], and two studies (6.2%) in lower-middle-income countries [71,72]. No studies were published in a low-income country.

### 3.2. Quality Assessment

**Figure 2:**
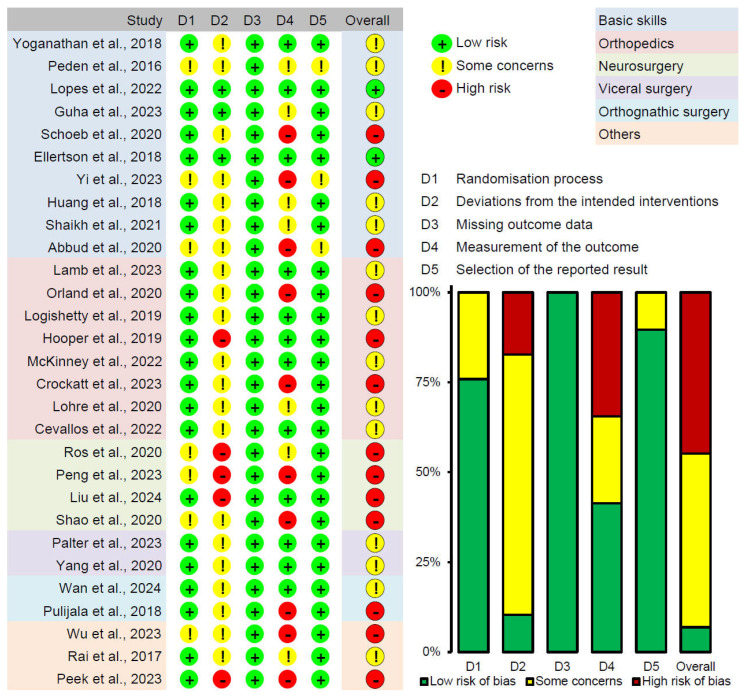
Risk of bias assessment for individual studies (randomized parallel design) and summary using the ROB-2 tool [44,47,48,49,50,51,53,54,55,56,57,58,59,60,61,62,63,64,65,66,67,68,69,70,71,72,74,75,76].

**Figure 3:**
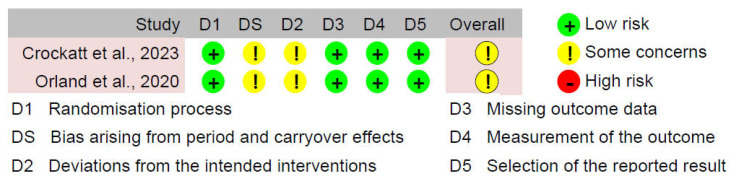
Risk of bias assessment for randomized crossover studies using the ROB-2 tool [45,46].

**Figure 4:**
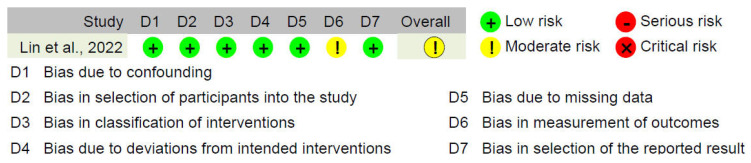
Risk of bias assessment for a non-randomized study using ROBINS-I tool [52].

**Figure 5:**
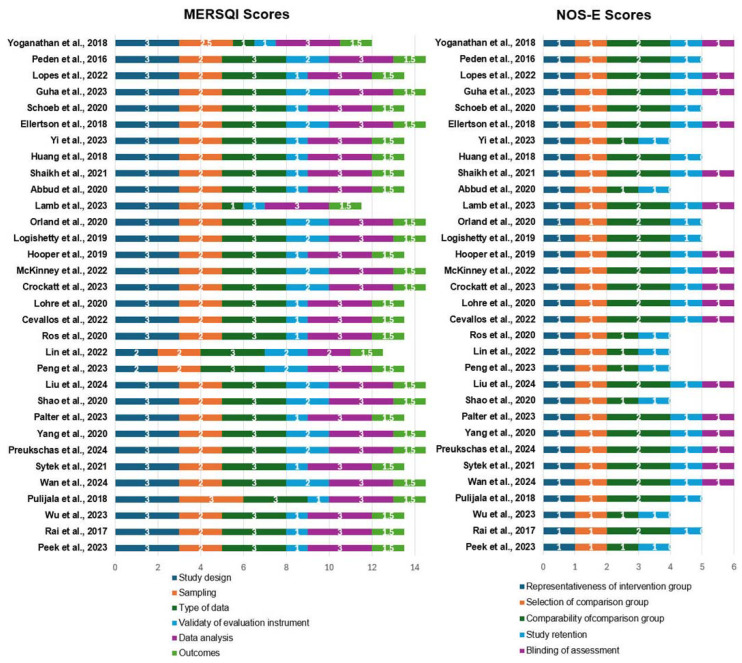
MERSQI and NOS-E scores of included studies [44,45,46,47,48,49,50,51,52,53,54,55,56,57,58,59,60,61,62,63,64,65,66,67,68,69,70,71,72,74,75,76].

The quality and bias of the included studies were assessed using four evaluation tools: ROB-2, ROBINS-I, MERSQI, and NOS-E. Analysis using the ROB-2 and ROBINS-I tools showed that the risk of bias primarily arose from intention-to-treat analysis and outcome assessment (see Figure 2, Figure 3 and Figure 4). Specifically, the most common sources of bias in randomized studies with parallel design (n = 29, 90.6%) were related to deviations from intended interventions (“D2” in Figure 2) and outcome assessment (“D4” in Figure 2). Among these studies, 26 (81.2%) had concerns or high risk in “D2”, and 17 (53.1%) had concerns or high risk in “D4”. This may be due to the unique aspects of ExR teaching, such as trainees wearing HMDs, which make it difficult to maintain absolute blinding for trainees, educators, or assessors regarding group assignments, even with strict blinding procedures. Additionally, in two randomized studies with crossover design (6.2%), period and carryover effects were an important source of bias (“DS” in Figure 3). Due to constraints related to the organization and timing of educational activities, these studies did not implement a washout period. Consequently, the traditional teaching methods and ExR teaching might have influenced each other, increasing the risk of bias. For the sole non-randomized study included (3.1%), in addition to inherent selection bias, the ROBINS-I assessment indicated potential bias arising from outcome assessment (“D6” in Figure 4). Nevertheless, the proportion of studies rated as high risk for bias (n = 13, 40.6%) did not exceed 50%, providing sufficient reliability for this review. The MERSQI and NOS-E scores were 13.72 ± 0.76 and 5.28 ± 0.85, respectively, indicating a generally high quality in study design, randomization, data analysis, and blinding methods (see Figure 5).

### 3.3. Surgical Education Content

#### 3.3.1. Education Theme

The included studies encompassed themes ranging from basic skills to specialized techniques (see Table 3). Ten studies (31.2%) focused on basic surgical skills [48,50,51,58,59,61,62,66,72,74], such as knot tying [58], basic suturing [62,74], central venous catheter (CVC) placement [50], bladder catheterization [51,66], emergency chest drainage [48], and arteriotomy and closure [59]. Additionally, these studies included basic laparoscopic skills such as peg transfer, circle cutting, needle guidance, lifting and grasping, clip application, and suturing with intracorporeal knot tying [61,72].

Twenty-two studies (68.8%) emphasized specialized techniques. For instance, eight studies (25.0%) focused on orthopedic surgery [44,45,46,49,60,63,70,75], covering procedures such as intramedullary nailing, arthroplasty, and pinning of the slipped capital femoral epiphysis. Five studies (15.6%) emphasized neurosurgical training, presenting techniques that include external ventricular drainage (EVD), aneurysm localization, and skull base tumor resection [52,53,54,57,67]. Visceral surgery was addressed in three studies (9.4%) [61,65,68], highlighting specialized procedures such as cholecystectomy [65,68] and liver surgery [76]. Three studies (9.4%) underscored the significance of imaging and anatomical comprehension during orthognathic surgical training [47,55,71], such as surgical planning for Le Fort I osteotomy and bilateral sagittal split ramal osteotomy (BSSRO). Other specialized techniques (9.4%) covered include spinal nerve block [51], bioptic indirect ophthalmoscopy (BIO) [64], and advanced life support protocols for post-cardiac surgery [69].

**Table 3:** Surgical education content involved in studies included.

Citation	Surgical Educational Content
***Basic surgical skills (n = 10)***	
Yoganathan et al. [58]	Single-handed reef knot tying
Peden et al. [62]	Basic interrupted suture placement training
Lopes et al. [74]	Five types of sutures for basic surgical skills
Guha et al. [59]	Practical skills training for arteriotomy and closure
Schoeb et al. [66]	Bladder catheter placement training
Ellertson et al. [51]	Bladder catheterization instruction
Yi et al. [48]	Surgery training on pneumothorax and chest tube drain management
Huang et al. [50]	Central venous catheter placement training
Shaikh et al. [72]	Laparoscopic surgical skills training focusing on intracorporeal knot tying
Abbud et al. [61]	Laparoscopic urological skills training including peg transfer, circle cutting, and needle guidance
***Orthopedics (n = 8)***	
Lamb et al. [44]	Tibia intramedullary nail surgery, covering steps, instrumentation, and proper techniques
Orland et al. [45]	Procedural training for tibial intramedullary nail insertion using a synthetic bone model
Logishetty et al. [60]	THA focusing on acetabular component orientation
Hooper et al. [70]	THA focusing on anatomy, imaging, and mechanical alignment
McKinney et al. [75]	Medial unicompartmental knee arthroplasty with Zimmer Persona system
Crockatt et al. [46]	Reverse total shoulder arthroplasty emphasizing augmented baseplate implantation
Lohre et al. [63]	Reverse shoulder arthroplasty training, emphasizing technique and decision making
Cevallos et al. [49]	Pinning of the slipped capital femoral epiphysis, covering anatomy and pin placement
***Neurosurgery (n = 5)***	
Ros et al. [67]	Free-hand EVD training covering theoretical knowledge and operational accuracy
Lin et al. [52]	Training for Kocher’s point localization and free-hand EVD, focusing on operational accuracy
Peng et al. [53]	Free-hand EVD and hematoma puncture procedures, focusing on operational accuracy
Liu et al. [54]	Anatomy of the intracranial vascular tree and localization of aneurysms
Shao et al. [57]	Procedures for skull base tumors, covering theory, diagnosis, and surgical methods
***Visceral surgery (n = 3)***	
Palter et al. [65]	Technical skills of laparoscopic cholecystectomy for effective execution
Yang et al. [68]	Laparoscopic cholecystectomy procedures, emphasizing dissection, clipping, and extraction
Preukschas et al. [76]	Surgical liver anatomy and decision making
***Orthognathic surgery (n = 3)***	
Sytek et al. [47]	Orthognathic surgery training focusing on surgical planning
Wan et al. [55]	Bimaxillary orthognathic procedure emphasizing surgical strategy and step sequences
Pulijala et al. [71]	Le Fort I osteotomy instruction with anatomy, tools, and step sequences
***Other procedures (n = 3)***	
Wu et al. [56]	Posterior medial branch block for lumbar facet joint syndrome
Rai et al. [64]	Binocular indirect ophthalmoscopy for retinal diagnosis and surgery
Peek et al. [69]	Advanced life support protocols for post-cardiac surgery scenarios, including shock defibrillation and emergency resternotomy

THA = total hip arthroplasty; EVD = external ventricular drainage.

#### 3.3.2. Target Audience

The primary audience for these basic skills training sessions consisted of medical or nursing students (undergraduates and graduates), whereas the training in specialized techniques targeted not only students but also junior or senior residents.

#### 3.3.3. Organization of Activities

Most included studies (n = 30, 93.8%) employed a cluster design [44,45,46,48,49,50,51,52,53,54,55,56,57,58,59,60,61,62,63,64,65,66,67,68,69,70,71,72,74,75], while two (6.2%) used a crossover design [47,76]. Thirty-one studies (96.9%) claimed to use randomization methods [44,45,46,47,48,49,50,51,53,54,55,56,57,58,59,60,61,62,63,64,65,66,67,68,69,70,71,72,74,75,76]. In the cluster design, the main strategy was to use “traditional teaching methods” as a control group, with “ExR-assisted education” as the intervention. However, two studies (6.2%) included an additional intervention group combining both traditional and ExR-assisted teaching [45,62]. In the crossover design, all trainees received both traditional and ExR methods but in different sequences. To ensure trainees were suitable candidates for the study and educational activities, half of the studies (16/32, 50%) reported arranging pre-training in knowledge or skills for all participants [45,46,49,50,54,55,56,59,60,63,67,68,69,70,74,76].

#### 3.3.4. Traditional Teaching Methods

The included studies’ traditional teaching methods encompassed theoretical and practical instruction. Theoretical teaching included standardized lectures [48,56,60,64], introductory presentations [55,56,69,74], and case-based or problem-based seminars [57]. Learning materials used included textbooks [52,70], surgical technique guides [44,45,46,49,59,67,69,70,75], anatomical or imaging atlases [52], and multimedia resources such as slides [71,74], pre-recorded 2D surgical videos [49,50,52,55,57,58,63,68,69], medical imaging data on monitors [54,56,61,76]. Practical teaching involved on-site surgical observation [52], demonstration [52,62], and mentorship during actual surgeries performed in collaboration with experienced surgeons [65]. It also included simulation exercises using commercial surgical models or cadaver specimens [46,47,48,50,51,59,62,64,66,69,74], where trainees could either explore independently [46,59] or receive face-to-face expert guidance and feedback during the procedures [50,51,62,64,66,69,74]. In the study by Schoeb et al. [51], group learning was organized where non-operating individuals of the group acted as peer evaluators, providing feedback to the operating student.

#### 3.3.5. ExR-Assisted Teaching Methods

**Figure 6:**
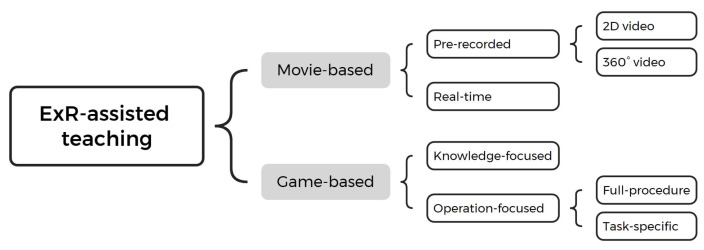
A taxonomy of ExR-assisted teaching.

Compared to traditional surgical teaching, ExR-assisted teaching offered further diverse and engaging methods. Based on the trainees’ mode of participation, ExR-assisted teaching can be categorized into “game-based” and “movie-based” types (see Figure 6). The “game-based” type represents a high level of interactivity, where trainees influence the progression and outcome of the “surgical simulation game” by controlling characters, moving objects, or making decisions. In contrast, the “movie-based” type is a relatively passive media form where trainees can only watch the development of the “surgical story” without directly affecting its course.

Most studies involved “game-based” ExR, yet it is notable that a few used the movie-based type [50,54,58,59,62,67,74,76]. For instance, Lopes et al. used AR glasses to display standard 2D instructional videos for CVC placement, allowing trainees to watch and practice simultaneously [50]. Yoganathan et al. and Guha et al. showed trainees 360° videos of reef knot tying [58] and suture placement [59] through VR-HMDs, while in the study by Ros et al. [67], trainees watched the complete EVD procedure in VR, learning through the “eyes of an expert”. Unlike pre-recorded videos, in the study by Rai et al. [74], trainees received real-time guidance from remote instructors via HMD.

In the “game-based” studies, a small portion focused on surgical knowledge [54,57,67,76], typically creating interactive environments where trainees could view 3D anatomical models of human organs or medical images, observe pathological changes, or explore different surgical approaches. The majority, however, concentrated on training operational skills, including specific surgical procedures, instrument handling, and hand–eye coordination.

Based on the completeness of the simulation process, these training methods could be divided into two subtypes: full procedure simulations and task-specific simulations. Full procedure simulations emphasize learning step sequences, often conducted in highly immersive environments with a modular approach. This is commonly seen in training for complex surgeries such as orthopedic or orthognathic procedures involving plenty of steps and instruments [44,45,46,47,49,51,52,55,63,65,68,69,70,71,75]. For example, the VR training system introduced by Wan et al. achieved full procedural simulation from intubation anesthesia to the completion of surgery [55]. Trainees must select and place the correct instruments in the correct locations to trigger the next steps. Task-specific simulations, on the other hand, focused on the deliberate practice of one or two critical steps within a surgery [48,53,56,60,61,64,66,72]. This approach is often used for minor surgeries or basic surgical skills training. For instance, the study by Peng et al. specifically emphasized the accuracy of target puncture during the EVD procedure [53], while Logishetty et al. focused on angular alignment of acetabular component orientation during total hip arthroplasty (THA) [60].

#### 3.3.6. Assessment of Education

Assessment is crucial to educational activities to confirm learning outcomes, provide feedback, and improve teaching methods. Assessments can be categorized by timing into baseline assessment, immediate post-assessment, and delayed post-assessment. Baseline assessments are conducted before the start of the training to understand the trainees’ initial levels and confirm their suitability for the educational and research activities. Immediate post-assessments were carried out immediately after the training to evaluate the trainees’ acquisition of new knowledge and skills. Delayed post-assessments, conducted weeks or months after the training, aim to assess the long-term effectiveness of the teaching, including retention and transfer of knowledge and skills. While all studies included in this review performed immediate post-assessments, half (n = 16, 50%) conducted baseline assessments [44,45,46,47,48,51,54,59,60,63,65,66,68,70,71,72]. Studies that included delayed post-assessments were even rarer (n = 2, 6.2%) [51,67], with only one claiming to have conducted all three types of assessments [51].

The assessment measures employed in the included studies could be categorized into knowledge, skills, and attitude assessments.

##### Knowledge Assessment

Twelve studies conducted knowledge assessments [46,48,52,54,56,57,63,67,68,70,71,76], which included written [46,67,70,71], oral [51,52,57,63,68], or computerized exams [54,76]. These assessments typically quantified the speed [63,76], correctness, or completeness of the trainees’ answers, evaluating their understanding of various surgical techniques, methods, and procedures, as well as foundational knowledge such as anatomy and imaging [54,76].

##### Skills Assessment

Twenty-four studies (75.0%) assessed skills through practical exams [56,58,59,60,61,62,64,66,74], simulated surgeries [44,45,46,47,49,50,52,53,55,63,69,70,72,75], or supervised actual operations [65]. Key quantitative indicators included task performance (e.g., the success rate of the task [45,46,51,55,58,59,61,62,64,65,66,70,74,75]), adherence to procedural norms (e.g., avoiding breaches of sterile principles [51,66] or dangerous maneuvers [44,45,55,63,69,72,75]), precision in operations (e.g., operational deviation [47,49,52,55,56,60,63]), and proficiency (e.g., operation time [45,47,49,50,52,55,56,59,64,69,70,72,74,75]).

##### Attitude Assessment

**Figure 7:**
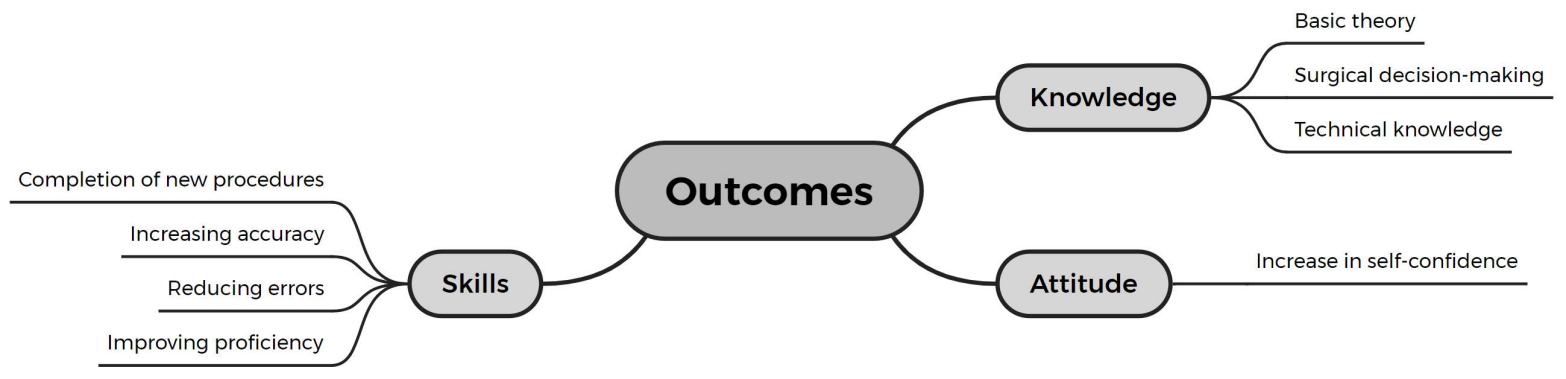
Educational outcomes of surgical training using ExR-HMDs.

Six studies (18.8%) evaluated changes in trainees’ attitudes [53,62,63,64,66,71]. Self-assessment of confidence was the most common topic and format for these evaluations [53,62,63,64,66,71], reflecting the effectiveness of the training and improvements in professional competence (see Figure 7).

Notably, eleven studies (34.4%) utilized standardized assessment tools [46,47,59,63,65,66,70,71,72,74,76]. In terms of skills assessment, these tools included assessment standards accredited by medical associations [47,76], and Global Rating Scales (GRS), such as the Objective Structured Assessment of Technical Skill (OSATS) [77], the Objective Structured Clinical Examination (OSCE) [78], and the Ottawa Surgical Competency Operating Room Evaluation (O-SCORE) [79]. Bandura’s self-efficacy scale was used for attitude assessment. The use of standardized assessment tools ensured stable and impartial measurement results [71].

### 3.4. The Pedagogy

#### 3.4.1. Educational Outcomes

##### Knowledge Outcomes

Among the twelve studies (37.5%) that conducted knowledge assessments [46,48,52,54,56,57,63,67,68,70,71,76], seven reported improvements in knowledge acquisition within the ExR group [48,54,56,57,67,76], particularly in basic theory (e.g., anatomical knowledge), surgical decision making, and technical knowledge (see Figure 7). For instance, Yi et al. found that the VR group showed significantly greater improvement in understanding pneumothorax and chest tube management compared to the traditional group [48]. Liu et al. noted that the AR group performed better in preoperative planning for aneurysm surgery and understanding anatomical structures, demonstrating the ability to internalize and transfer this knowledge by showing improved comprehension and application even without AR assistance during image interpretation [54]. Additionally, Ros et al. reported that the VR group exhibited better short-term knowledge acquisition than the traditional group and maintained superior knowledge retention six months later [67]. However, five studies (15.6%) did not observe significant superiority of the VR group in knowledge acquisition or reported only marginal advantages [46,52,68,70,71]. Notably, Lin et al. concluded the opposite, finding that traditional teaching methods (textbooks) were more effective in delivering theoretical knowledge about EVD surgery [52].

##### Skills Outcomes

Among the 24 studies (75.0%) that conducted skills assessments [44,45,46,47,49,50,52,53,55,56,58,59,60,61,62,63,64,65,66,69,70,72,74,75], 21 found that ExR methods were superior or at least advantageous in one aspect compared to traditional methods, demonstrating significant benefits in surgical education [44,45,47,49,50,52,53,55,56,58,59,60,61,63,64,65,66,69,70,72,74]. These advantages included successfully completing new procedures, enhancing accuracy, reducing errors, and improving proficiency. Only three studies found no advantages of ExR over traditional methods (see Figure 7) [46,57,62].

*Completion of New Procedures*: Yoganathan et al. reported that the VR group learned single-handed knot tying faster and had a higher task completion rate than the control group [58]. Orland et al. [45] found that the VR group had a higher completion rate for tibial IMN surgery. Rai et al. observed that AR trainees demonstrated higher proficiency in bioptic indirect ophthalmoscopy (BIO) operations earlier than those in the traditional group [64]. Guha et al. noted that MR training improved vascular surgery skills more effectively, particularly in selecting appropriate surgical instruments and performing procedures [59]. Abbud et al. reported significant differences in learning outcomes for laparoscopic suturing tasks between the MR and traditional groups, with MR users showing a steeper learning curve and quicker skill acquisition [61].

*Increasing Accuracy*: Sytek et al. highlighted VR’s significant advantages in the depth and precision of surgical planning [47]. Logishetty et al. found that in THA acetabular component positioning, the AR group had a smaller average orientation error (1° ± 1°) compared to the traditional group (6° ± 4°), suggesting that AR technology might be more effective in providing real-time feedback and enhancing spatial awareness [60]. Compared to the control group, Peng et al. reported significant improvements in the MR group’s operational precision, puncture depth control, and puncture accuracy [53].

*Reducing Errors*: Wan et al. found that the VR group made fewer tool selection errors during Le Fort I osteotomy than the control group [55]. Lamb et al. noted that the VR group required fewer corrections during tibial intramedullary nailing (IMN) surgery, indicating a potential advantage of VR training in reducing operational errors, albeit the difference was small [44].

*Improving Proficiency*: Lamb et al. reported that VR trainees performed better in terms of completion time for simulated surgeries, indicating that VR training can effectively improve surgical efficiency [44]. Lopes et al. found that the AR group had a faster overall speed in performing independent suturing [74]. Wu et al. observed significant improvements in the MR group’s performance in spinal nerve block procedures, particularly in reducing the number of puncture attempts and the time taken [56].

Nonetheless, some studies still indicated that ExR did not consistently outperform traditional methods across all skill dimensions. For example, Peek et al. reported that while the VR group was slower in performing surgical tasks, they were more accurate and made fewer errors [69]. This suggests that traditional physical training might have advantages in speed for emergency situations requiring rapid responses. Cevallos et al. found that in slipped capital femoral epiphysis (SCFE) fixation training [49], the VR group showed a significant advantage only in the angle deviation of the needle relative to the growth plate but performed similarly to the traditional group regarding task completion time, number of attempts, and avoiding improper operations.

##### Attitude Outcomes

Different studies have reached varying conclusions regarding the impact of ExR on trainees’ self-confidence (see Figure 7). Both Peng et al. [53] and Pulijala et al. [71] found a significant increase in self-confidence among the ExR group. Specifically, Pulijala et al. conducted a longitudinal comparison of self-confidence improvement before and after training in Le Fort I osteotomy, concluding that the ExR group’s improvement was more pronounced than that of the traditional group [71]. On the other hand, despite the advantages in learning experience and skill performance, four studies (12.5%) suggested that ExR may not necessarily be superior to traditional methods in enhancing self-confidence [62,63,64,66]. For instance, Schoeb et al. reported that the traditional group exhibited greater improvement in confidence regarding theoretical knowledge of bladder catheterization [66].

#### 3.4.2. The Theories

Nine studies (28.1%) cited well-known teaching theories or paradigms in their publications (see Table 4). Six of these studies (18.8%) claimed to have applied these theories or paradigms in designing, organizing, and evaluating their educational activities. For instance, in task design for instructors, both Yoganathan et al. and Guha et al. emphasized using Peyton’s four-step approach in their studies [58,59], ensuring that both the VR and traditional groups followed this method for teaching single-handed knot tying and basic arteriotomy and closure techniques. Regarding task design for trainees, Palter et al. [65] applied Ericsson’s concept of deliberate practice within the VR training system. Trainees were required to repeatedly practice specific tasks until they achieved a sufficient level of proficiency, aiming to strengthen skills in areas where they showed weaknesses. Orland et al. integrated the theory of spaced repetition into VR simulation training by scheduling training sessions 3-4 days apart, with the goal of enhancing learning outcomes and long-term retention of information [45]. In evaluations, Guha et al. utilized the VARK model (Visual, Auditory, Reading/Writing, Kinesthetic) during baseline testing to understand trainees’ learning styles and explore their impact on the effectiveness of MR-assisted learning [59]. Pulijala et al. developed a self-confidence scale for trainees based on Bandura’s social cognitive theory and the concept of self-efficacy [71]. This scale covered various confidence elements necessary for residents, aiming to assess the impact of VR training on self-efficacy.

Four studies (12.5%) cited teaching theories when interpreting and discussing their results. For instance, Lamb et al. referenced psychomotor theory to explain the benefits of VR in tibial IMN training compared to the traditional group [44]. The immersive environment allowed trainees to focus more on the surgical procedure itself rather than environmental factors or other distractions, thereby developing muscle memory and operational proficiency more quickly. Ros et al. cited the mirror neuron theory in the educational context to explain the advantages of the first-person perspective in VR immersive environments for skill acquisition [67]. The authors argued that learning “through the expert’s eyes” involves a lower cognitive load than learning “from the other side.” Liu et al. used Kolb’s experiential learning theory to explain why the AR group performed better in aneurysm localization tasks [54]. Trainees could observe and reflect on the differences between their performance and the expert standard through the “expert’s eyes,” understand their mistakes and shortcomings, and improve through repeated practice and feedback. This process helped them form concrete concepts and strategies from abstract theories and observations, enhancing their spatial reasoning skills. Conversely, Yang et al. used chunking learning theory to explain the “opposite” findings, where the traditional group (video) outperformed the VR group in planning subsequent surgical steps [68]. The coherent video presentations in the control group helped trainees build a comprehensive knowledge structure, making planning the next steps smoother and more intuitive. In contrast, the VR group trained in a fragmented learning environment, where each step appeared as a separate “chunk” of information that needed to be individually learned and practiced.

### 3.5. The Technology

#### 3.5.1. HMDs and User Interaction

Twenty-one studies (65.6%) reported using VR [44,45,46,47,48,49,51,52,55,57,58,63,65,67,68,69,70,71,72,75,76], six (18.8%) used AR [50,54,60,62,64,74], and five (15.6%) used MR technology [53,56,59,61,66]. Regarding HMDs, 19 (59.4%) utilized general-purpose devices: seven (21.9%) used Microsoft HoloLens [53,56,57,59,60,61,66], six (18.8%) used Oculus Rift [47,51,55,70,71,76], and six studies (18.8%) each used Oculus Quest [69], Magic Leap [54], Vuzix [74], Brother AiRScouter [50], Google Glasses [62], and HTC Vive Pro [52]. Ten studies (31.2%) employed specialized devices from medical extended reality companies: four (12.5%) used Osso VR [44,45,49,75], two (6.2%) used Precision OS [46,63], two (6.2%) used LapSim VR [65,72], and two studies (6.2%) each used LapMentor and EyeSI BIO [64,68]. Three studies did not report the specific names of the HMDs used [48,58,67].

The HMDs can be categorized based on their design: monocular glasses (Brother AiRScouter [50]), binocular glasses (Vuzix [74] and Google Glasses [62]), see-through helmets (HoloLens [53,56,57,59,60,61,66] and Magic Leap [54]), and non-see-through helmets [44,45,46,47,49,51,55,63,64,65,68,69,70,71,72,75,76]. In terms of usage, 21 studies (65.6%) used HMDs as standalone devices [44,45,46,47,49,51,52,53,55,56,57,59,60,61,63,66,69,70,71,75,76], while in several studies, HMDs were used as non-standalone display devices within training systems, such as neuronavigation systems [54], laparoscopic systems [65,68,72], or for viewing VR-formatted videos on smartphones [58,67].

Regarding user interaction methods, HoloLens users could interact directly with virtual objects using voice or gestures [53,56,57,59,60,61,66]. Other interaction methods included using control handles [44,45,46,47,49,51,55,63,70,71,75,76] or haptic feedback devices [51,52] to interact with the training systems.

#### 3.5.2. Benefits and Drawbacks

**Figure 8:**
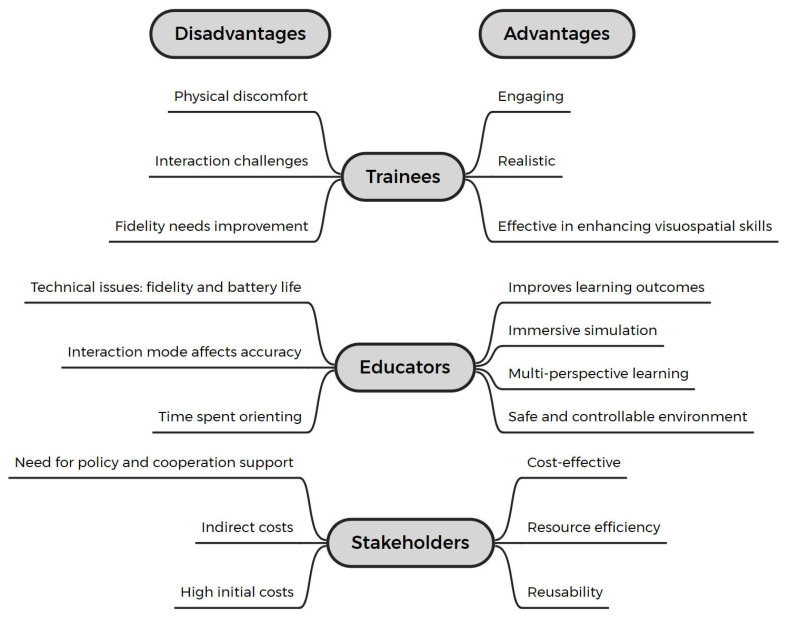
Trainee, educator, and stakeholder perspectives on the advantages and disadvantages of using ExR-HMDs.

In the included studies, trainees, educators, and stakeholders provided insights into the advantages and disadvantages of using HMDs (see Figure 8).

##### Perspectives from Trainees

Twenty studies (62.5%) reported conducting the user experience (UX) surveys with trainees [44,46,47,48,50,51,52,53,54,56,57,59,60,61,62,66,69,70,74,76], employing methods such as Likert-scale questionnaires and interviews. Seventeen studies (53.1%) presented their survey results, indicating that the majority of trainees had an overall positive attitude towards ExR technology. Trainees found ExR to be engaging, realistic, easy to use, comfortable, and effective in enhancing visuospatial skills.

However, some trainees expressed contrary or skeptical views on certain aspects. For instance, in the studies by Preukschas et al. and Logishetty et al. [60,76], some trainees felt that the system’s fidelity needed improvement, commenting that the “virtual platform’s replication of reality was average” and that there was “no significant improvement compared to traditional simulation methods.” In Sytek et al.’s study, some trainees faced challenges while interacting with the platform [47]. They noted that “the VR system was extremely sensitive to user inputs, causing minor gestures or movements to result in significant reactions in the virtual environment.” They reported feeling frustrated when making necessary fine adjustments. Similarly, in Huang et al.’s study, participants complained that the extra effort required for fine adjustments extended the overall training time [50]. Additionally, some trainees experienced discomfort using ExR-HMDs, reporting headaches, dizziness, and fatigue in the VR study by Yi et al. and the MR study by Guha et al. [48,59].

##### Perspectives from Educators

As reflected in the studies, educators’ perspectives largely suggested that IEs were effective in improving learning outcomes and were more effective than traditional teaching methods (see Section 3.4). ExR offered advantages not found in traditional methods, such as immersive simulation, multi-perspective learning, and intuitive spatial understanding. Compared to cadaver-based courses, ExR created a “safe and controllable training environment, allowing trainees to practice repeatedly until mastery” [46]. Additionally, this immersive environment provided trainees with “high-quality educational experiences, increasing student engagement” [59], and “reduces distractions, thus enhancing focus” [67]. However, some studies noted that the immersive environment “caused students to spend more time orienting themselves within the system” [51], and the mode of interaction influenced the accuracy of operations [59]. Some researchers also reported technical limitations of ExR systems, such as fidelity, where improvements in haptic feedback are needed [45,46,47,55,70], and the need to simulate soft tissue responses during operations [60]. Other technical concerns include system fluidity [74] and HMD battery life [59,66].

Despite these issues, nearly all researchers remain optimistic about the future application of ExR technologies. Seven studies suggested that ExR should be viewed as a complement to traditional teaching methods rather than a replacement [45,51,52,54,55,67,69].

##### Perspectives from Stakeholders

Nineteen studies (59.4%) reported perspectives from the stakeholders, i.e., policy and support organizations [46,49,51,52,53,54,55,56,59,60,62,63,64,67,68,71,72,74,76], primarily focusing on the cost, cost-effectiveness ratio (CER), and policy and cooperation support related to ExR-HMDs compared to traditional teaching methods.

Six studies (18.8%) mentioned that ExR-HMD technology is less expensive than commercial surgical simulators [52,54,55,60,63,67]. Logishetty et al. reported that the procurement cost of an ExR teaching system is approximately one-tenth of that of commercial simulators [60]. Three studies indicated that the ability of ExR to facilitate repeated practice significantly reduced consumable costs [46,49,63]. Lohre et al. conducted quantitative calculations to estimate the advantages of VR technology in improving the CER [63]. The authors found that every hour of VR training could save 48 min of actual operating room training time, increasing the CER by 34.1 times. Additionally, the reusability of VR for different trainees could enhance the CER by up to 685 times [63]. Crockatt et al. noted that VR allowed trainees to practice repeatedly, easily covering routine surgeries or rare and special cases, significantly reducing the costs of using cadaver specimens (e.g., purchase, preservation, and large-scale professional facilities) [46].

However, three studies (9.4%) noted that the price and setup costs of ExR can still be relatively high for some institutions, potentially challenging the budgets of certain hospitals or universities [56,64,71]. Some studies documented cases where self-developed ExR systems were used for surgical teaching, achieving results with smaller financial budgets. Nevertheless, this increased content creation costs, development time, specialized software, and staff training [53,67,74,76]. Lopes et al. defined these as indirect costs, which, though difficult to quantify precisely, should not be ignored [74]. In this regard, six studies (18.8%) highlighted the importance of policy and cooperation support for promoting ExR. For example, policy support for free software can accelerate the creation of teaching content [67,74]. Strengthening cooperation among medical education institutions, technology providers, and support organizations can help integrate educational, technical, and financial resources, which, over time, will gradually reduce costs and promote the adoption of ExR [59,62]. It is necessary to identify and promote technologies that are most likely to add value, and research the return on investment, including economic returns, long-term skill retention, and patient care improvements [51,64].

## 4. Discussion

This systematic review explores the application of ExR-HMDs in surgical education, providing a comprehensive summary of the current state of development and differing views in this field. The results indicate that ExR-HMDs are effective in surgical education and offer advantages not found in traditional surgical education methods. In some instances, ExR-HMDs are at least as effective as traditional methods. ExR-HMDs create engaging, safe, and controlled immersive training environments that achieve the educational goals of knowledge and skill acquisition and potentially enhance students’ confidence and interest in learning.

The characteristics of the included studies suggest that although the application of ExR in surgical education is increasing, there is an uneven geographical and economic distribution. The majority of these studies are conducted in high-income countries, where most of the HMDs used in the research are also developed and manufactured. Access to these technologies appears to be challenging in middle-income countries, and no studies from low-income countries could be included. This discrepancy warrants attention, as countries with less developed infrastructures have been considered significant beneficiaries of ExR technologies. However, various structural challenges, such as a lack of funding, low levels of industrialization, underdeveloped surgical education systems, and immature interdisciplinary collaboration frameworks, may exacerbate the technical difficulties of implementing ExR technologies in these regions. Therefore, conducting more ExR pilot studies in these countries and regions is crucial to better understand the technical barriers and develop more accessible and scalable ExR technologies to address these challenges.

In high-income and upper-middle-income countries, ExR-HMDs offer certain cost advantages, particularly when compared to traditional commercial simulators. The initial investment is generally lower, and the ability to reuse the technology significantly reduces long-term costs. However, challenges remain in content creation, equipment procurement, and operational costs, especially for educational institutions with limited budgets. Over the long term, with policy support, collaboration, and the use of free development software, ExR-HMD technology can become an efficient and economical training method. These measures can help mitigate the high costs associated with developing and maintaining ExR systems, making them more accessible and sustainable for widespread adoption.

The following paragraphs first address each AQ from the three dimensions separately: content, pedagogy, and technology. Subsequently, the dimensions are integrated to provide a comprehensive perspective, where educational content is used as the context, and the advantages and disadvantages of ExR-HMD technology are analyzed from the perspective of pedagogical theories.

Regarding the content (AQ 1), the included studies addressed various educational topics, from basic surgical skills to specialized techniques. Basic skills such as knot tying, suturing, and catheter placement were frequently covered, while specialized techniques included orthopedic, neurosurgical, and visceral procedures. The activities mostly involved interactive simulations, where trainees practiced these skills in a controlled, immersive environment.

Regarding the pedagogy (AQ 2), ExR-HMDs demonstrated positive educational outcomes. Most studies reported improvements in knowledge acquisition, skill development, and increased confidence among trainees. These technologies often enhanced accuracy, reduced errors, and improved proficiency compared to traditional methods. However, a few studies indicated that traditional methods could still be effective in certain contexts. Integrating pedagogical theories such as Peyton’s four-step approach, Ericsson’s deliberate practice, and Kolb’s experiential learning helped design effective educational interventions. These theories provided a strong foundation for the instructional strategies used and helped to interpret the effectiveness of ExR-HMD training.

Regarding the technology (AQ 3), the primary benefits noted were increased immersion, improved spatial awareness, and enhanced interaction. User interactions were facilitated through diverse methods like voice commands and gestures. However, challenges such as system fidelity, operational inconvenience, and physical discomfort were reported. Technical improvements are needed, particularly in haptic feedback and soft tissue simulation. Despite these challenges, there is optimism about integrating ExR technologies with traditional teaching methods to enhance surgical education.

Surgical education is a meticulously designed and implemented educational process to guide, facilitate, and support surgical trainees in acquiring foundational knowledge, operational skills, and professional attitudes. This process leads to relatively enduring changes in behavior and behavioral potential as a result of simulation training and clinical practice experiences. Effective surgical education should positively change knowledge, skills, and attitudes. As demonstrated in the included studies, these changes can be assessed by comparing pre- and post-training performance. Specifically, improvements can be seen in trainees’ deep understanding and application of surgical knowledge, operational skills, proficiency enhancement, and more advanced and mature professional attitudes (e.g., increased self-confidence).

The “See One, Do One, Teach One” theory proposed by American surgeon William Halsted in 1889 has profoundly impacted surgical education [87,88]. This approach emphasizes mastering surgical skills through observation, practice, and teaching. However, this method faces several challenges in practical educational settings, including resource limitations and significant pressure on trainees. In terms of resources, trainees often lack sufficient access to cases or cadavers, leading to limited practice opportunities. The practical resources relied upon by traditional methods, such as operating rooms and materials, are also highly constrained. Additionally, trainees face considerable psychological pressure, as they need to quickly master skills with limited practice, often resulting in a lack of confidence due to insufficient training. ExR-HMD technology offers significant improvements in these areas. First, ExR-HMD can create highly realistic virtual surgical environments, allowing trainees to engage in repeated, even unlimited, simulation practice without the constraints of real cases and resources, thereby greatly enhancing resource utilization efficiency. Second, this technology provides real-time feedback and diverse simulation scenarios, enabling trainees to continuously practice and refine their skills in a safe, risk-free environment, thus reducing the psychological pressure associated with making mistakes. Through these advancements, ExR-HMD not only increases the accessibility of training resources but also effectively alleviates the pressure on trainees during the learning process, representing innovative progress in surgical education.

“Students never come to the classroom with empty heads” is a classic analogy from Piaget’s constructivist educational theory [89]. Similarly, rarely do surgical trainees enter the laboratory or operating room as blank slates. They actively incorporate new content into their existing knowledge frameworks, integrating prior experiences with new information. ExR-HMD technology, particularly in game-based skill training, enhances the interactivity of the learning environment. Trainees can engage actively through human–machine interaction and simulated operations. The repetitive practice, trial and error, and adjustments during these operations provide immediate feedback, facilitating the assimilation and accommodation of knowledge. Moreover, the immersive scenarios created by ExR-HMD can realistically replicate surgical environments to varying degrees, thereby enhancing the learning experience. This immersion helps trainees apply and expand their knowledge frameworks in real-world contexts. The included studies also indicate that the training scenarios created by ExR can effectively prepare future surgeons by demonstrating the necessary preparations and strategies.

Piaget’s constructivist educational theory emphasizes that learners actively construct new knowledge based on their existing knowledge. Vygotsky’s sociocultural theory further posits that learning occurs within the zone of proximal development (ZPD) through interactions and communication with more experienced individuals, such as teachers or peers [90,91]. This review found that using ExR-HMD for surgical education is not always significantly superior to traditional teaching methods in terms of knowledge improvement. Nearly half of the 12 studies that assessed knowledge reported comparable or better outcomes with traditional methods. This can be explained through Vygotsky’s theory, which highlights the importance of social interaction in knowledge construction. In traditional teaching methods, instructors’ and students’ interactions, discussions, and feedback are crucial for promoting deep understanding. In ExR-based knowledge training, the lack of interaction with experienced surgeons or mentors may limit learners’ comprehension and application of complex concepts. Additionally, the effectiveness of training can be impacted if it does not appropriately target the learner’s ZPD. Training that remains within the “comfort zone” may fail to challenge the trainee adequately, while too difficult training may lead to frustration and disengagement. Therefore, it is crucial to balance the difficulty of tasks to keep them within the ZPD, where trainees are optimally challenged and supported. To enhance the effectiveness of ExR in knowledge training, the authors suggest integrating ExR with traditional teaching methods. Specifically, foundational knowledge can be introduced and discussed through traditional methods, followed by applying this knowledge and practicing skills using ExR. This combination can achieve a more effective integration of knowledge and skills.

In surgical education, professional attitudes are often overlooked. A surgeon’s confidence is a professional attitude that does not arise from temporary or incidental emotional reactions but is continually developed through training and practice [92]. Confidence can directly influence a surgeon’s performance, aiding in appropriate responses under pressure. In contrast, a lack of confidence in junior surgeons can lead to minor or significant errors due to feelings of being overwhelmed [93]. Surgeons’ confidence can originate from two sources: intrinsic sources, through sufficient training and effort, and extrinsic sources, from psychological support and encouragement provided by educators. ExR technologies enhance surgeons’ mastery of skills by offering high-intensity, high-frequency simulation practices, such as deliberate practice, thereby promoting intrinsic confidence. Traditional education, on the other hand, strengthens the psychological support system through mentorship, feedback, and emotional support, enhancing extrinsic confidence. Reflected in the studies included, ExR and traditional education may be equally effective in boosting surgeons’ confidence. Therefore, combining the strengths of ExR and traditional education can simultaneously enhance skill training and psychological support. This comprehensive approach could significantly boost surgeons’ confidence, ensuring their development in both technical and psychological aspects.

While ExR-HMD teaching is generally promising, not all research findings support its flawless effectiveness. Some studies have reported technical shortcomings and issues encountered during use, such as system fidelity, operational inconvenience, and physical discomfort. These issues can hinder educational activities and impact overall effectiveness. It is important to consider that trainees from diverse backgrounds and experiences may perform differently when adapting to and using ExR-HMDs. Those lacking prior experience with the technology might require additional time and effort to become accustomed to the system, including learning basic operational methods, interaction techniques, and device characteristics, potentially leading to initial confusion and discomfort. For example, the technical difficulty of precisely adjusting virtual objects might cause frustration or passivity during the learning process, thus affecting educational outcomes. Moreover, inadequate task design and physical discomfort can increase trainees’ fear, resistance, or distrust of new technology. However, as technology continues to evolve, future advancements are likely to improve both user performance and user experience, addressing these challenges and enhancing the overall effectiveness of ExR-HMD in surgical education.

The studies included in this systematic review scored well in quality assessments using MERSQI and NOS-E. Bias analysis indicated that the risk of bias primarily stemmed from intention-to-treat analysis and outcome assessment. This may be due to the distinct nature of ExR teaching compared to traditional methods (e.g., trainees wearing HMDs), which makes it challenging to ensure that trainees, educators, or evaluators remain unaware of group assignments despite rigorous blinding procedures.

Researchers acknowledged limitations in their studies, including (1) limited sample sizes, (2) the representativeness of trainees, which might affect the generalizability of the conclusions to a broader population, and (3) the lack of long-term longitudinal observations to explore the retention of knowledge and skills.

Furthermore, only 9 out of the 32 included studies (28.1%) cited educational theories or paradigms to guide their research design, evaluation, or interpretation of results. This highlights an opportunity for future research to strengthen its theoretical foundation. Studies can enhance the scientific rigor and consistency of their design and evaluation by more systematically incorporating educational theories.

In addition to the limitations of the included evidence, this systematic review itself has methodological limitations. First, the broad scope encompassing various educational activities and ExR technologies led to significant heterogeneity among the studies. To address this heterogeneity, the review employed qualitative synthesis and narrative review methods, which, while providing detailed descriptions and analyses, may lack the quantitative summary and overall effect estimates that meta-analyses offer. Second, this review focused on the effectiveness of ExR-HMD in surgical education, emphasizing educational and learning outcomes. Consequently, studies that did not address these outcomes were excluded, even if they met other criteria, and reported on user experience or satisfaction. Third, all studies involving HMDs in the intervention group were included without considering the proportion of ExR within the educational activities. Finally, only English-language publications were included, potentially excluding relevant studies in other languages.

Despite these limitations, this systematic review provides a comprehensive overview of the current state of ExR-HMD technology in surgical education. It highlights areas for future research and development, suggesting that with further refinement and integration of educational theories, ExR-HMD has the potential to significantly enhance surgical training outcomes worldwide.

## 5. Conclusions and Recommendations

This systematic review revealed that ExR-HMD technology is generally effective in surgical education, offering advantages not found in traditional methods. However, its application is primarily concentrated in high-income countries, with middle-income countries facing challenges in access, and no studies from low-income countries were included. This disparity underscores the need for targeted efforts to make ExR-HMD technology more accessible globally.

ExR-HMDs create engaging, immersive training environments that enhance knowledge and skill acquisition, potentially boosting trainees’ confidence and interest in learning. Despite these benefits, some studies reported technical issues such as system fidelity, operational inconvenience, and physical discomfort, which can impact educational outcomes. Additionally, the effectiveness of ExR-HMDs in knowledge training may be limited if social interactions with mentors are lacking.

Combining ExR-HMD technology with traditional teaching methods is recommended to address these challenges. This approach can leverage both strengths, ensuring comprehensive skill training and psychological support for trainees. Future research should focus on integrating educational theories more systematically to enhance the design and evaluation of ExR-HMD interventions. Furthermore, efforts should be made to conduct more pilot studies in middle- and low-income countries to understand and overcome technical and economic barriers.

For future research directions, it is important to focus on refining the educational content and curricula specifically designed for ExR-HMDs. Tailoring content to maximize the advantages of immersive technologies will ensure more effective and engaging learning experiences. Additionally, conducting longitudinal studies would be advantageous to evaluate the long-term effects of this technology on the skills and performance of surgical trainees in real-world practice, which could provide valuable insights into the retention of knowledge and skills over time and the sustained benefits of ExR-HMD-enhanced training.

In conclusion, while ExR-HMD technology significantly enhances surgical education, further refinement and integration with traditional methods are needed. This systematic review provides valuable insights and recommendations for future research and development, aiming to make ExR-HMD technology a standard, accessible tool in surgical training worldwide.

## Figures and Tables

**Table 1:** A summary of systematic reviews on extended reality-based head-mounted displays (ExR-HMDs) in medical education.

Reference †	Time Span [Year]	Technology Type *	Scope of Education	Review Focus *
Barteit et al., 2021 [26]	2014–2019	VR, AR, and MR-based HMD	Medicine	The effectiveness, advantages, and disadvantages
Berthold et al., 2022 [27]	2000–2020	VR-based HMD	Orthopedics	The efficacy
Gsaxner et al., 2023 [28]	2016–2021	AR and MR-based HMD (only HoloLens)	Medicine	The application and effectiveness
Tursø-Finnich et al., 2023 [29]	Up to 2020	VR-based HMD	Medicine	The usage, advantages, and disadvantages

† Sort by first author’s last name (A–Z). * Different systematic reviews may have defined concepts variously. AR = augmented reality; ExR = extended reality; HMD = head-mounted display; MR = mixed reality; VR = virtual reality.

**Table 2:** PICOS framework for inclusion and exclusion criteria.

Category	Description	Inclusion	Exclusion
Population	Healthcare personnel receiving surgical education	Surgical team members (e.g., surgeons, nurses) and medical students	Non-healthcare personnel (e.g., patients, technicians)
Intervention	Use of ExR-HMDs either in whole or as part in a surgical educational context	All HMDs (e.g., helmet, smart-glasses) and ExR types (virtual, augmented, and mixed reality)	(a) Non-HMDs (e.g., projectors, smartphones, tablets); (b) surgery-uninvolved education (e.g., non-clinically oriented anatomical education); or (c) non-educational context (e.g., preoperative conversations, surgical planning, intraoperative navigation)
Comparison	ExR-HMDs vs. traditional methods for surgical education	All kinds of traditional methods (e.g., observational learning, lectures, textbook, cadaveric dissection, animal simulation, video demonstrations)	The counterpart was not a traditional method, or there was no comparison
Outcome	Improvement in learning outcomes	Concrete learning outcome or evaluation of the effectiveness of ExR-HMDs	No concrete learning outcome or evaluation reported
Study	Original and experimental studies published in English	Literature identified and screened, regardless of publication status	Non-English literature; non-original studies (e.g., reviews or meta-analysis); non-empirical studies (e.g., pipelines, technical notes) without validation purposes

PICOS = Population, Intervention, Comparison, Outcome, Study.

**Table 4:** Surgical education theories or paradigms involved in studies included.

Theory or Paradigm	Description	Involved Studies
Peyton’s four-step approach [80]	A structured method for teaching procedural skills involving demonstration, deconstruction, comprehension, and performance.	Yoganathan et al. [58], Guha et al. [59]
Deliberate practice (Ericsson 2004) [81]	Focuses on repeated practice and feedback to improve performance, particularly in areas where trainees show weaknesses.	Palter et al. [65]
Spaced repetition [82]	A learning technique that involves repeating training sessions with intervals in between to enhance long-term retention and learning outcomes.	Orland et al. [45]
VARK modalities (Fleming 1987) [83]	A framework for understanding learning styles based on four modalities: Visual, Auditory, Reading/Writing, and Kinesthetic (VARK).	Guha et al. [59]
Social cognitive theory (Bandura 2006) [84]	Emphasizes the role of observational learning, social experience, and self-efficacy in behavior change and skill acquisition.	Pulijala et al. [71]
Psychomotor theory (Fitts and Posner 1967) [21]	Explains how motor skills are acquired through the integration of cognitive and physical processes, often enhanced by immersive environments.	Lamb et al. [44]
Mirror neuron theory	Suggests that observing an action activates the same neural pathways as performing the action, facilitating learning and skill acquisition through imitation.	Ros et al. [67]
Experiential learning (Kolb 2014) [85]	A model of learning through experience involving a cyclical process of concrete experience, reflective observation, abstract conceptualization, and active experimentation.	Liu et al. [54]
Chunking learning (Chase and Simon 1973) [86]	Describes how information is better understood and remembered when it is organized into coherent, manageable chunks rather than fragmented pieces.	Yang et al. [68]

## Data Availability

The data presented in this study are available upon reasonable request from the corresponding authors.

## Supplementary Materials

The following supporting information can be downloaded at: https://www.mdpi.com/article/10.3390/bioengineering11080741/s1, File: PRISMA 2020 Checklist [94].

## Abbreviations

The following abbreviations are used in this manuscript:

2D	Two-Dimensional
3D	Three-Dimensional
AR	Augmented Reality
AQ	Analytical Questions
BIO	Bioptic Indirect Ophthalmoscopy
BSSRO	Bilateral Sagittal Split Ramal Osteotomy
CER	Cost-Effectiveness Ratio
CVC	Central Venous Catheter
EVD	External Ventricular Drainage
ExR	Extended Reality
GRS	Global Rating Scales
HMD	Head-Mounted Display
IMN	Intramedullary Nailing
MERSQI	Medical Education Research Study Quality Instrument
MR	Mixed Reality
NOS-E	Newcastle–Ottawa Scale-Education
OSATS	Objective Structured Assessment of Technical Skill
OSCE	Objective Structured Clinical Examination
O-SCORE	The Ottawa Surgical Competency Operating Room Evaluation
PICOS	Population, Intervention, Comparison, Outcome, Study
PRISMA	The Preferred Reporting Items for Systematic reviews and Meta-Analyses
RoB 2	Risk Of Bias tool for randomized trials
ROBINS-I	Risk Of Bias In Non-randomized Studies - of Interventions
SCFE	Slipped Capital Femoral Epiphysis
SWiM	Synthesis Without Meta-analysis
THA	Total Hip Arthroplasty
TPACK	Technological Pedagogical Content Knowledge
VARK	Visual, Auditory, Reading/Writing, and Kinesthetic
UX	User Experience
VR	Virtual Reality
ZPD	Zone of Proximal Development

## Appendix A

The Preferred Reporting Items for Systematic reviews and Meta-Analyses (PRISMA) 2020 checklist (see Supplementary Material File).

## Appendix B. Search Terms and Strings

### Appendix B.1. Search Terms

**Table A1:** Search terms.

Main Concept	Thesaurus	Grammatical Variants *
Extended reality	augmented reality	—
	extended reality	—
	mixed reality	—
	virtual reality	—
Surgery	operation	operability, operable, operate, operated, operation’s, operational, operative, operatively, operatives, operator, operator’s operators
	procedure	procedures, procedural, procedurally, procedure’s
	surgery	surgeries, surgical, surgery’s
Education	education	educability, educable, educates, educational, education’s, educators, educator’s, educative, educate, educated, educating, educations
	learning	learn, learned, learning’s, learnings, learns
	teaching	teaches, teach, teachings, teaching’s
	training	train, train’s, trained, training’s, trainings, trains

* “—” represents exact matching of the term, without including grammatical variants during actual retrieval.

### Appendix B.2. Search String for Each Data Source

**Table A2:** Search String for Each Data Source.

Data Source	Search String
PubMed	(“virtual reality” OR (“virtual Reality”[MeSH Terms]) OR “augmented reality” OR (“augmented Reality”[MeSH Terms]) OR “mixed reality” OR “extended reality”) AND (((surg* OR operat* OR procedur*) AND (educat* OR teach* OR train* OR learn*)) OR (“education, medical”[MeSH Terms])) AND (2014/1/1:3000/12/12[pdat]))
Cochrane Library	#1 (“virtual reality”):ti,ab,kw
	#2 MeSH descriptor: [Virtual Reality] explode all trees
	#3 (“augmented reality”):ti,ab,kw
	#4 MeSH descriptor: [Augmented Reality] explode all trees
	#5 (“mixed reality”):ti,ab,kw OR (“extended reality”):ti,ab,kw
	#6 #1 OR #2 OR #3 OR #4 OR #5
	#7 (surg*):ti,ab,kw OR (operat*):ti,ab,kw OR (procedur*):ti,ab,kw
	#8 (educat*):ti,ab,kw OR (teach*):ti,ab,kw OR (train*):ti,ab,kw OR
	#9 #7 AND #8
	#10 MeSH descriptor: [Education, Medical] explode all trees
	#11 #9 OR #10
	#12 #6 AND #11 Filter: Publication date: 01/01/2014—01/04/2024
Web of Science (Core) ^a^	(TS =“virtual reality” OR TS = “augmented reality” OR TS = “mixed reality” OR TS = “extended reality”) AND (TS = surg* OR TS = operat* OR TS = procedur*) AND (TS = educat* OR TS = teach* OR TS = train* OR TS = learn* ) AND (PY = 2014–2024)
ScienceDirect	#1 tak(“virtual reality”) AND (tak(surgery) OR tak(operation) OR tak(procedure)) AND (tak(education) OR tak(teaching) OR tak(training) OR tak(learning))
	#2 tak(“augmented reality”) AND (tak(surgery) OR tak(operation) OR tak(procedure)) AND (tak(education) OR tak(teaching) OR tak(training) OR tak(learning))
	#3 tak(“mixed reality”) AND (tak(surgery) OR tak(operation) OR tak(procedure)) AND (tak(education) OR tak(teaching) OR tak(training) OR tak(learning))
	#4 tak(“extended reality”) AND (tak(surgery) OR tak(operation) OR tak(procedure)) AND (tak(education) OR tak(teaching) OR tak(training) OR tak(learning))
	#5 Merge #1, #2, #3, and #4 Filter: Year: 2014—2024
Scopus	( TITLE-ABS-KEY ( “virtual reality” ) OR TITLE-ABS-KEY ( “augmented reality” ) OR TITLE-ABS-KEY ( “mixed reality” ) OR TITLE-ABS-KEY ( “extended reality” ) ) AND ( TITLE-ABS-KEY ( surg* ) OR TITLE-ABS-KEY ( procedur* ) OR TITLE-ABS-KEY ( operat* ) ) AND ( TITLE-ABS-KEY ( educat* ) OR TITLE-ABS-KEY ( teach* ) OR TITLE-ABS-KEY ( train* ) OR TITLE-ABS-KEY ( learn* ) ) AND PUBYEAR > 2013 AND PUBYEAR < 2025
ACM Digital Library	“query”: { (Abstract:(“virtual reality”) OR Abstract:(“augmented reality”) OR Abstract:(“mixed reality”) OR Abstract:(“extended reality”)) AND (Abstract:(surg*) OR Abstract:(procedur*) OR Abstract:(operat*)) AND (Abstract:(educat*) OR Abstract:(teach*) OR Abstract:(train*) OR Abstract:(learn*)) } “filter”: { E-Publication Date: (01/01/2014 TO *), ACM Content: DL }
IEEE Xplore	((“Abstract”:“virtual reality”) OR (“Abstract”:“augmented reality”) OR (“Abstract”:“mixed reality”) OR (“Abstract”:“extended reality”)) AND ((“Abstract”:surg*) OR (“Abstract”:operat*) OR (“Abstract”:procedur*)) AND ((“Abstract”:educat*) OR (“Abstract”:teach*) OR (“Abstract”:train*) OR (“Abstract”:learn*)) Filter: Year: 2014—2024
Google Scholar	(“virtual reality” OR “augmented reality” OR “mixed reality” OR “extended reality”) AND (surg* OR operat* OR procedur*) AND (educat* OR teach* OR train* OR learn*) Filter: Year: 2014—2024; First 200 records in best match
WorldCat	(kw:“virtual reality” OR kw:“augmented reality” OR kw:“mixed reality” OR kw:“extended reality”) AND (kw:surg* OR kw:operat* OR kw:procedur*) AND (kw:educat* OR kw:teach* OR kw:train* OR kw:learn* ) Filter: Year: 2014—2024; Format: Articles; First 100 records in best match

^a^ TS represents topic searches among title, abstract, and keywords.

### Appendix B.3. Search Results

**Table A3:** Results count per data source on identification stage.

Data Source	Search Results Count *	Exported Results Count
PubMed	3935	3935
Cochrane Library	1324	1324
Web of Science (Core)	4981	4981
ScienceDirect	93	93
Scopus	13,184	13,184
ACM Digital Library	278	278
IEEE Xplore	1272	1272
Google Scholar	17,500	200
WorldCat	9772	100
Total	52,339	25,367

* Retrieve date: 30 March 2024.
